# DAT1 Polymorphism Determines L-DOPA Effects on Learning about Others’ Prosociality

**DOI:** 10.1371/journal.pone.0067820

**Published:** 2013-07-04

**Authors:** Christoph Eisenegger, Andreas Pedroni, Jörg Rieskamp, Christian Zehnder, Richard Ebstein, Ernst Fehr, Daria Knoch

**Affiliations:** 1 Behavioral and Clinical Neuroscience Institute, Department of Psychology, University of Cambridge, Cambridge, United Kingdom; 2 Division of Social and Affective Neuroscience, Department of Psychology, University of Basel, Basel, Switzerland; 3 Center for Economic Psychology, Department of Psychology, University of Basel, Basel, Switzerland; 4 Faculty of Business and Economics, Quartier UNIL-Dorigny, University of Lausanne, Lausanne, Switzerland; 5 Department of Psychology, National University of Singapore, Singapore, Singapore; 6 Laboratory for Social and Neural Systems Research, Department of Economics, University of Zurich, Zurich, Switzerland; University G. D’Annunzio, Italy

## Abstract

Despite that a wealth of evidence links striatal dopamine to individualś reward learning performance in non-social environments, the neurochemical underpinnings of such learning during social interaction are unknown. Here, we show that the administration of 300 mg of the dopamine precursor L-DOPA to 200 healthy male subjects influences learning about a partners’ prosocial preferences in a novel social interaction task, which is akin to a repeated trust game. We found learning to be modulated by a well-established genetic marker of striatal dopamine levels, the 40-bp variable number tandem repeats polymorphism of the dopamine transporter (DAT1 polymorphism). In particular, we found that L-DOPA improves learning in 10/10R genoype subjects, who are assumed to have lower endogenous striatal dopamine levels and impairs learning in 9/10R genotype subjects, who are assumed to have higher endogenous dopamine levels. These findings provide first evidence for a critical role of dopamine in learning whether an interaction partner has a prosocial or a selfish personality. The applied pharmacogenetic approach may open doors to new ways of studying psychiatric disorders such as psychosis, which is characterized by distorted perceptions of others’ prosocial attitudes.

## Introduction

Finding generic prosocial interaction partners and distinguishing them from selfish ones is of major importance in our social and economic well-being. People learn about a partner’s prosocial preferences by gathering information either through personal interactions or by using information about the reputation of the interaction partner [1]. When external information about someone’s prosocial preferences is not available, one has to learn this, via trial and error, in repeated interactions with the partner [2]. However, strategic motives may overcast such learning, as they create an incentive for selfish partners to appear prosocially in order to be able to profit from future interactions.

Despite the fact that learning about a partners’ prosocial preferences is a fundamental aspect of our everyday social lives, little is yet known about the regional neurochemical systems that influence learning in social contexts. So far, basic research in non-human animals [3] and human neuroimaging studies using drug challenges [4], [5], as well as studies with individuals suffering from Parkinson’s disease [6], [7], [8] have delineated the dopamine system with a particular emphasis on the striatum as being a fundamental basic neurocircuitry underlying probabilistic reward-learning in humans. However, recent research has begun to probe the involvement of the striatum in more complex behaviors typically observed in repeated social interactions between two individuals. For instance, human neuroimaging studies investigating the neural correlates of repeated trust interactions have shown that positive social feedback such as reciprocated trust activates an individual’s striatum, whereas selfish, non-reciprocated trust leads to a decrease in striatal activity [1], [2], for a review see [9]. Furthermore, activation in the striatum also predicts future trust decisions [10], suggesting that striatal activity might signal the rewards of positive social feedback and thereby guides future decisions. Thus, it appears that reward learning based on social outcomes (e.g., social approval, positive emotional responses and positive social feedback in repeated interactions) is coded similarly in reward circuitry as if feedback was based on non-social outcomes [11], [12], [13]. In sum, there is much reason to believe that a pharmacological manipulation of striatal dopamine modulates learning about others’ prosocial preferences by relying fundamentally on a basic probabilistic reward-learning mechanism.

Striatal dopamine levels are dependent on the availability of the dopamine transporter (DAT) protein, as it reuptakes dopamine from the synaptic cleft into the pre-synaptic compartment after its release. Therefore, DAT is an important regulator of dopamine signaling, most primarily in the striatum, as it only occurs in low concentrations in other areas of the brain [14]. There is substantial genetic variation in protein expression levels, and this variation is assumed to affect endogenous striatal dopamine levels. The most extensively studied gene variant in this context is the 40 base-pair variable number tandem repeat polymorphism of the dopamine transporter (DAT1 polymorphism) [15]. Basic neurobiological research has shown that the 9-repeat (9R) variant of the DAT1 polymorphism is associated with lower transporter protein expression than the 10R variant [16], [17]. Neurochemical imaging research in humans reported a lower density of dopamine transporter in striatum of individuals who carry a 9/10R genotype (heterozygotes) compared to those who carry the 10/10R (homozygotes) genotype [18]. Hence, 9/10R genotype individuals are expected to have higher extrasynaptic striatal dopamine levels than 10/10R carriers [19], [20], [21], [22]. Accordingly, functional imaging studies have consistently reported that 9/10R genotype carriers show greater activity in the striatum during processing of rewards compared to 10/10R carriers [19], [20], [21]. Data stemming from Parkinson’s disease patients who are treated with L-dihydroxy-phenylalanine (L-DOPA, a biochemical precursor of dopamine) suggest that the drug interacts with the DAT1 polymorphism in ways that are consistent with the above line of arguments. Patients who carry the 9/10R genotype are more likely to experience long term side-effects of L-DOPA treatment, which can be linked to high levels of dopamine in the striatum, compared to those who carry the 10/10R genotype [23].

Here we explore how L-DOPA induced increases in brain dopamine levels interact with genetically determined individual differences in endogenous striatal dopamine levels to influence learning about a partners’ prosocial preferences. We administered 300 mg of L-DOPA to 205 subjects, who were all genotyped for their DAT1 polymorphism (Materials S1). As L-DOPA is mainly converted to dopamine in the striatum [24], endogenous striatal dopamine levels might interact with exogenous administration of L-DOPA to influence net dopamine levels [23]. Based on this line of arguments, we test the hypothesis that the effects of L-DOPA administration on learning about others’ prosociality depends on an individual’s DAT1 polymorphism. A pharmacogenetic approach [25], [26] allows a specific interpretation of the observed effects, i.e. in the present context whether the DAT1 polymorphism is predictive of the direction of the effects of a pharmacological challenge on reward learning.

## Materials and Methods

### Subjects

205 healthy young Swiss males with mean (SD) age of 23.5 years (3.6) took part in our double-blind, parallel group and placebo controlled experiment. The study was performed in accordance with the Declaration of Helsinki and approved by the Cantonal Ethic Commission Zurich. Subjects had no significant general psychiatric, medical, or neurological disorder based on the result of structured interviews; they were included in the study after having provided written informed consent. Three subjects were excluded due to self-reported nausea, and two because they did not understand the instructions.

### Genotyping

The polymorphism for the DAT-1 was characterized using PCR amplification procedure with the following primers:

DAT-1:

F5′-TGTGGTGTAGGGAACGGCCTG-3′.

R5′-CTTCCTGGAGGTCACGGCTCA-3′.

PCR reactions were performed using 5 µl Master Mix (Thermo scientific), 2 µl primers (0.5 µM), 0.6 µl Mg/Cl2 (2.5 mM), 0.4 µl DMSO 5% and 1 µl of water to total of 9 µl total volume and an additional 1 µl of genomic DNA was added to the mixture. All PCR reactions were employed on a Biometra T1 Thermocycler (Biometra, Güttingem, Germany). PCR reaction conditions were as follows:

Preheating step at 94.0°C for 5 min, 34 cycles of denaturation at 94.0°C for 30 s, reannealing at 55°C for 30 s and extension at 72°C for 90 s. The reaction proceeded to a hold at 72°C for 5 min. All reaction mixtures were electrophoresed on a 3% agarose gel (AMRESCO) with ethidium bromide to screen for genotype.

### Subject Grouping According to DAT1 Polymorphism

The 9/10R and the 10/10R genotypes accounted for the majority of the observed genotypes in our sample (48% and 44%, respectively, Table 1), and we used these two genotypes throughout the analyses. The system was in Hardy-Weinberg equilibrium. The observed and expected heterozygosity were 0.88 and 0.79 respectively.

**Table 1:** DAT1 polymorphism allele frequencies in our sample.

DAT1 polymorphism genotypes	Placebo	L-DOPA	Total
10/10R	45	51	96
9/10R	46	42	88
9/9R	8	3	11
9/11R	1	2	3
7/10R	1	0	1
10/11R	0	1	1
Total	101	99	200

### Experimental Procedure

Subjects were randomly assigned to receive either a single dose of 300 mg of Madopar (consisting of 300 mg L-DOPA and 75 mg benserazide, a peripheral dopa-decarboxylase inhibitor) or a placebo. They then received a standardized meal and 100 ml of water. On the evening before the experiment and 30 min before L-DOPA administration, subjects were required to ingest 10 mg of domperidone in order to avoid possible peripheral dopaminergic side effects such as nausea and orthostatic hypotension. After subjects had read the instructions, we checked whether they had understood the rules of the game by providing control questions. All but two of the subjects answered these control questions correctly. Subjects performed the task 50 min after L-DOPA intake. The task was implemented in z-Tree software and presented on computer screens [27]. Subjects were also requested to perform a mouthwash to collect buccal epithelial cells for the preparation of DNA. All subjects received a flat fee of CHF 100 for participation in the experiment and an additional payment according to the points earned in the task. Each point earned was worth CHF 0.07. Each subject received payment in cash in private at the end of the experiment, based on the points earned.

### Experimental Design

In our paradigm, two players, player A and player B, begin with an endowment of 10 monetary units (MUs). First, player A has to decide how much of his endowment he wants to transfer to player B, knowing that the transfer is tripled by the experimenter. The transfer has an 80% probability of reaching player B. In this case, B can choose to either make a repayment that equalizes payoffs, or to retain the entire amount. The transfer is “lost” in the remaining 20% of the cases, so that player B receives nothing and cannot make a repayment. Thus, in case of an omitted return, player A does not know with 100% certainty whether this was player B’s intention.

To be able to observe learning over time, we let our subjects in the role of player A play several rounds of the task. Each player A plays 20 rounds of the task paired with the same player B in all rounds. Since an omitted return is an extremely powerful aversive social signal, we implemented the “lost transfer” possibility to avoid the problem that player As might immediately withhold positive transfers after observing a single non-repayment. All our subjects in the main experiment are in the role of player A. They are paired with player Bs for whom repayment decisions were pre-recorded, i.e. player Bs decided in how many of a total of 20 rounds they were going to make a repayment. Thus, player Bs made decisions in line with their true prosocial preferences. Player As were aware of the fact that they were paired with a player B whose decisions had been pre-recorded and also about the possibility that their transfers might get “lost” in 20% of the cases.

The use of pre-recorded player B decisions is necessary to avoid an important confound. If player A would interact simultaneously with a given player B they could vary transfers strategically to influence player B’s future behavior [28]. Specifically, by conditioning transfers on B’s previous repayments, A can generate reputational incentives for B to repay [29]. Thus, in repeated simultaneous interactions in this context a repayment is no longer a clear signal of a player B’s prosocial preferences, because a purely selfish player B may also repay due to reputational incentives and hide his or her true type [30]. To investigate player A’s pure learning process about a partners’ prosocial preferences within a reinforcement-learning framework, we eliminated these strategic elements by using pre-recorded decisions of player B. Player A could infer that repayments from player B can be interpreted as a pure signals of true prosocial preferences.

All player As in our main experiment were matched with one of two types of player Bs: a prosocial one, from whom player As received a repayment in 14 out of the 20 rounds, and a relatively selfish one from whom player As received a repayment in only 6 out of 20 rounds. Player As were not aware of the fact that we deliberately pre-selected a prosocial and a more selfish partner. All transfer decisions had real monetary consequences for player As, and they were told in the instructions that their decisions also have an influence on player Bs’ payoff, which retains the social aspect if this experimental setting. Furthermore, as player As do not have any information about the social preferences of player Bs at the outset, they have to rely on their everyday knowledge about how people would behave in such a social interaction situation. They can then use this information and learn, trial by trial, through positive or negative social feedback about player Bs’ prosocial preferences.

In sum, the fact that player As can not influence player Bs’ decisions allows us to exclude any strategic motives that might confound reward-learning behavior and allows to test in an clean way whether L-DOPA administration interacts with player As’ DAT1 polymorphism in modulating learning about a partner’s prosocial preferences.

### Pre-Recording of Player B Decisions

To pre-record the player Bs’ decisions, we conducted a session involving the same task design (without drug administration) several weeks before the main experiment. Each participant had to indicate in how many of 20 rounds he, in the role of player B, would make a repayment. After player B had decided how often he wanted to repay, the computer randomly distributed the repayment decisions across the 20 rounds of the experiment. This procedure allowed us to collect a large number of player B repayment decisions.

### Optimal Transfer Decision

Player A can choose transfers *x* ∈ [0,10]. Player B receives the transfer with probability of 0.8. In this case he can decide to retain all the money or to repay the amount of 2*x* to equalize payoffs. Player A’s transfer is lost with a probability of 0.2, meaning that player B cannot make a repayment. Player A’s optimal transfer *x* depends on the probability *p* with which player B repays when he receives the transfer. Player A’s expected profit E[π] is given as follows:
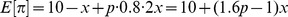



The expected profit is strictly increasing in *x* as long as *p*>5/8. Thus, if *p* is larger than 5/8, then player A profits most if he always transfers his whole endowment (that is, 10 MUs). If *p* is smaller than 5/8, then it is best to always transfer nothing (that is, 0 MUs). If *p* equals 5/8, player A is indifferent, as all possible transfers yield the same expected payoff. From this follows that profit-maximizing player As who are matched with prosocial player B should transfer their full endowment in each round, whereas player As who are matched with a selfish player B should not transfer anything.

### Measures of Drug Related Side Effects

Side effects were assessed using visual analog scales [31] and were recorded prior to substance administration and before the trust game was performed. Items in the scale were alert/drowsy, calm/excited, strong/feeble, muzzy/clear-headed, well coordinated/clumsy, lethargic/energetic, contented–discontented, troubled–tranquil, mentally slow/quick-witted, tense/relaxed, attentive/dreamy, incompetent/proficient, happy/sad, antagonistic/amicable, interested/bored and withdrawn/gregarious. These dimensions were presented as 10 cm lines on a computer screen and volunteers marked their current state on each line with a mouse click. In line with previous research [32], the factors “alertness”, “contentedness”, and “calmness” were calculated from these items.

### Statistical Analysis

Our statistical analysis is based on analysis of variance, Mann-Whitney tests and Spearman rank correlations. All tests are two-tailed tests. We examined the impact of partner type [with a binary indicator for partner type indicating whether the player A was confronted with the pre-recorded decisions of a prosocial player B ( = 1) or a ore selfish player B ( = 0)], L-DOPA [with a binary indicator for L-DOPA indicating whether the subject received L-DOPA ( = 1) or placebo ( = 0)], genotype [(with a binary indicator for subjects who carry a 9/10R genotype ( = 1) or a 10/10R genotype ( = 0)], and interactions between these variables in a univariate three-way ANOVA on the investor’s total earnings in the task. Further analyses included the reinforcement learning model parameters alpha and theta as dependent variables (Materials S1).

## Results

### Learning About a Partnerś Prosocial Preferences

**Figure 1:**
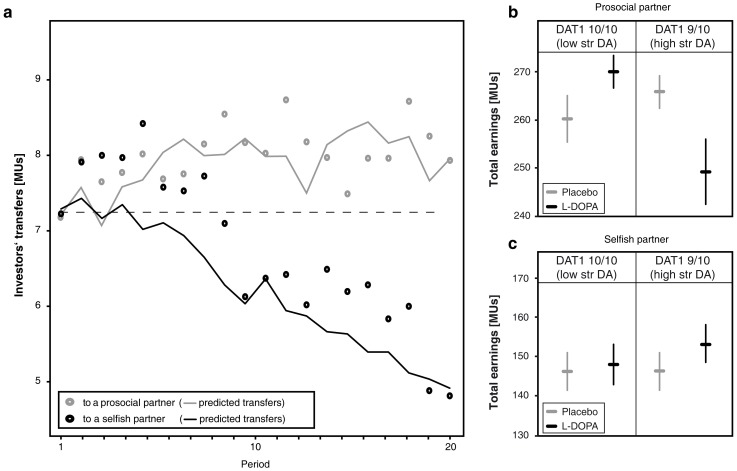
Average player As’ transfers across rounds and resulting earnings. (**a**) Player As’ transfers in each round during interactions with a prosocial (grey dots) respective selfish (black dots) player B over 20 rounds of the task. Player As increase their transfers over the 20 rounds when paired with a prosocial player B and decrease their transfers while interacting with a selfish player B. The learning curves represent the predicted transfers by the reinforcement learning model for interactions with a prosocial (grey line) and selfish (black line) partner. Hence, player As learn to adapt their transfers according to player Bs’ prosocial preferences. (**b/c**) Dopaminergic modulation of learning performance defined as the total earnings accumulated by player As. (**b**) When paired with a prosocial partner, player As who carry the 10/10R DAT1 genotype (lower striatal dopamine levels, n = 50) improve their learning performance under the influence of L-DOPA (placebo: n = 22; L-DOPA: n = 28). Player As who carry the 9/10R DAT1 genotype (higher striatal dopamine levels, n = 43) show an impaired performance after L-DOPA administration (placebo: n = 27; L-DOPA: n = 16). (**c**) Dopaminergic effects are absent when player As are paired with a selfish partner. Horizontal lines indicate average total earnings of player As, separately for L-DOPA and placebo groups and the 9/10R (placebo: n = 19; L-DOPA: n = 26) and the 10/10R DAT1 (placebo: n = 23; L-DOPA: n = 23) genotype carriers. Vertical lines indicate standard errors of the mean.

We found that subjects (total n = 200) successfully learn over time who is prosocial and who is not (Figure 1a). Average transfers increase over time when interacting with a prosocial partner (n = 101) and decrease with a selfish partner (n = 99). Analysis of transfers (in MUs) as the dependent variable using partner type (prosocial, selfish) as a between-subjects and rounds (1–20) as a within-subject factor showed a significant interaction effect of round×partner type (F(10.2,198)*_ = _*10.20, *p*<0.001, partial eta-square = 0.049). Transfers began to differ according to partner type, on average, by the ninth trial (Figure 1a: prosocial vs selfish type, Z_ = _3.00, *p*<0.003). Applying a standard reinforcement learning model [33] to the transfers revealed that the model adequately predicts player As’ learning behavior (Figure 1a, Materials S1, Figure S2).

### Dopaminergic Effects on Learning Performance

Having established that player As successfully learn about the prosocial preferences of their partners, we looked at the overall learning performance as measured by As’ total earnings in the task. The repayment probability of the pre-selected prosocial type ensured that transferring the full endowment (10 MUs) is the strategy maximizing expected profits (total earnings of 280 MUs), while the repayment probability of the pre-selected selfish type implied that transferring nothing would have maximized expected profits (total earnings of 200 MUs). We found that L-DOPA effects on earnings depend on DAT1 genotype and on partner type (interaction effect L-DOPA×DAT1×partner type on total earnings, F(1,176)* = *4.65, *p*<0.032, partial eta-square = 0.026). When subjects faced a prosocial partner, we found a significant interaction effect of L-DOPA×DAT1 genotype on learning performance (F(1,89) = 9.66, *p*<0.003, partial eta-square = 0.098). Specifically, L-DOPA increased learning performance in subjects carrying the 10/10R genotype, assumed to be associated with lower endogenous striatal dopamine levels, with placebo subjects earning an average of 260.2 MUs, while subjects on L-DOPA earned 270.9 MUs (Figure 1b: placebo vs L-DOPA in 10/10R genotype group, Z*_ = _*2.022, *p*<0.043). Conversely, we found that L-DOPA administration reversed this learning effect in those subjects carrying the 9/10R genotype, which is assumed to be associated with higher endogenous striatal dopamine levels, with placebo subjects earning 265.7 MUs and L-DOPA subjects earning an average of 249.3 MUs (Figure 1b: placebo vs L-DOPA in 9/10R genotype group, Z = 1.961, *p*<0.050). We found no dopaminergic effects on learning performance when investors faced a more selfish partner (Figure 1c: main and interaction effects of two-way ANOVA, all *p* values >0.454).

Because the observed pharmacogenetic effect on earnings might also result from player Aś level of prosociality before the task started (that is, their baseline prosociality), we checked whether there were differences across drug, player B type, and genotype in player Aś transfers in the first round of the task, but found no evidence for this (three-way ANOVAs, all *p* values >0. 202). Finally, controlling for side effects of L-DOPA administration using visual analogue scales, the reported interaction effect of L-DOPA×DAT1 on player As’ learning performance about interactions with a prosocial partner remains significant (F(1,86)_ = _7.76, *p*<0.007, partial eta-square = 0.083).

### Dopamergic Effects on Reinforcement Learning Parameters

The total earnings in our task reflect an important outcome variable of the player As’ learning process, but this measure says little about *how* learning takes place. To this end, we modeled player As’ learning within the framework of reinforcement learning [33]. The model employed here (Materials S1), disentangles two essential processes. The first process (captured by the model’s *learning rate* parameter) determines how strongly a given feedback from a player B (that is positive or no returns) changes the subjective value of the available transfer options (0–10 MUs). A low learning rate implies that the player Bs’ feedback has a relatively small impact on player As’ decision in the next round, whereas a high learning rate implies a relatively larger impact. The second process is captured by the *sensitivity* parameter. This parameter specifies the exploration-exploitation trade-off of reinforcement learning [34]. A high sensitivity parameter implies a strong focus on the transfer option with the highest subjective value, whereas a low sensitivity parameter implies that all transfer options will be chosen with substantial probability. For example, a given player A may assign the highest subjective value to the transfer of 10 MUs after a few interactions with a prosocial partner. If he persists with transferring 10 MUs for the remaining rounds, his sensitivity parameter would be high. If he continues by exploring alternative options (0–9 MUs), his sensitivity parameter would be low.

**Figure 2:**
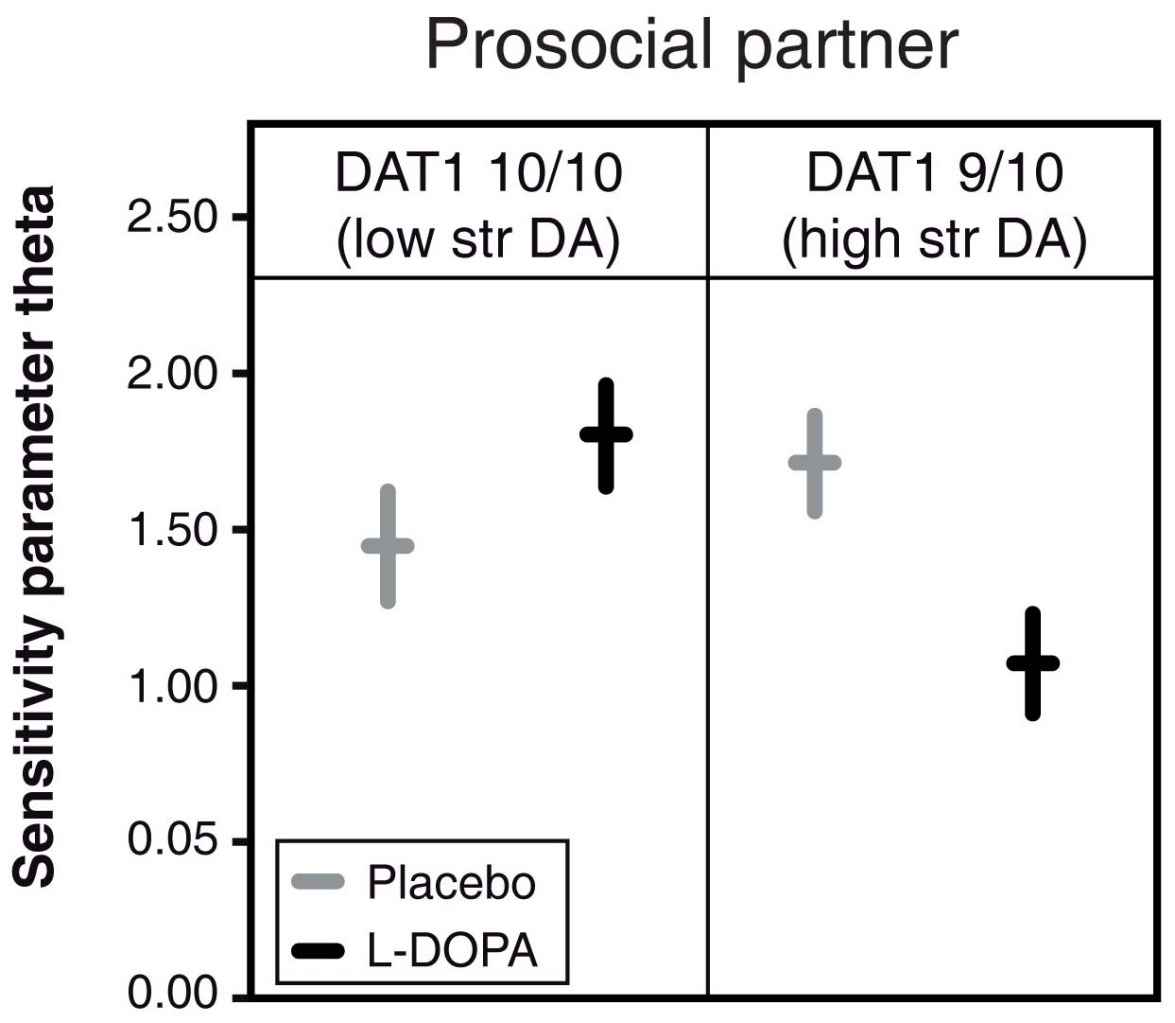
Pharmacogenetic effect on the sensitivity parameter. Administration of L-DOPA increases the sensitivity for making transfers that subjectively provide the largest expected return in player As who carry the 10/10R DAT1 genotype (lower endogenous striatal dopamine levels) (placebo: n = 22; L-DOPA: n = 28), but decreases this sensitivity in those who carry the 9/10R DAT1 genotype (higher endogenous striatal dopamine levels) (placebo: n = 27; L-DOPA: n = 16). Horizontal lines indicate mean values of the sensitivity parameter, separately for L-DOPA and placebo groups and the 9/10R and the 10/10R DAT1 genotype carriers. Vertical lines indicate standard errors of the mean.

In analogy to previous findings on reinforcement learning in non-social contexts [4], we did not observe any dopaminergic effects on the learning rate parameter (all *p* values >0.316). In contrast, we found a clear interaction of L-DOPA and player As’ DAT1 genotype on the sensitivity parameter (Figure 2: F(1,89)*_ = _*7.923, *p*<0.006, partial eta-square = 0.082). When expressing the sensitivity parameter for easier interpretation as the probability of choosing the transfer with the highest expected value (Figure S1), L-DOPA compared to placebo administration decreases the probability of choosing the transfer with the highest subjective value in 9/10R genotype carriers by 8.6 percentage points. On the other hand, this probability increases by 13.3 percentage points for 10/10R individuals following L-DOPA administration.

## Discusssion

We show that a manipulation of the dopaminergic system modulates learning about a partners’ prosocial preferences. In particular, we found that L-DOPA administration improves this learning process in carriers of the 10/10R genotype, which is assumed to be linked to lower endogenous striatal dopamine levels, but impairs learning in people who carry the 9/10R genotype, which is assumed to be linked to higher endogenous dopamine levels.

Our findings resemble an inverted-U shaped relationship between dopamine levels and learning performance that is that dopaminergic drugs such as L-DOPA might stimulate the dopaminergic system to optimal or overdosed levels in individuals with low vs. high baseline dopamine system functioning [35]. This may be understood in the context of theoretical and empirical accounts suggesting that optimal tuning of dopamine function in the prefrontal cortex [36], and as demonstrated recently also in the striatum [37], is vital for a variety of cognitive functions. In other words, there seems to be a critical range of dopamine stimulation for better behavioral performance, while behavioral performance above or below this critical range of dopamine stimulation deteriorates [38].

By modeling player As’ behavior within the framework of reinforcement learning we found no evidence of a dopaminergic modulation of the learning rate parameter, but found a clear interaction of L-DOPA and player As’ DAT1 genotype on the sensitivity parameter. Furthermore, the modeling results suggest that administering L-DOPA to investors who carry a 9/10R genotype confers lower earnings in the task by decreasing their sensitivity for exploiting the subjectively best transfer. In other words, it appears that the pharmacogenetic manipulation mostly affects the degree to which appropriately learned prosocial values of the other partner are used at the point of choice and not the capacity to update expectations on the basis of novel feedback during the learning phase. These results concur with recent computational models which emphasize a role for striatal dopamine in modulating the sensitivity parameter rather than the learning rate [39] and is also empirically supported by the finding that hyper-dopaminergic mice with a reduced expression of striatal DAT (DAT knockdown mice) display a diminished capacity to exploit learning opportunities, as represented by a lower value of their sensitivity parameter [40].

Our finding that individuals with highest striatal dopamine levels (9/10R carriers who received L-DOPA) benefit the least from transfers to a prosocial partner is also intriguing from a clinical perspective. Psychotic patients exhibiting paranoia show pronounced distrust of others, even though they reside in a conducive environment as part of their treatment regimen in the clinic. Current theories hold that psychosis might result from a disturbance in error-related updating of inferences and beliefs about the world [41] caused by an overactive mesolimbic dopaminergic system [42]. Indeed, a recent study found that psychotic individuals exhibit reduced reciprocal trust in response to positive feedback from a trustworthy trustee [43]. Thus, although speculative, these findings generally support our claim. Finally, our finding might have relevance for the pharmacological treatment of Parkinson’s disease, as patients who carry the 9/10R genotype are reported to face an increased risk of suffering from psychosis in response to L-DOPA treatment, a finding which was interpreted to result from overly high striatal dopamine levels [23].

The fact that we found no dopaminergic effects on learning performance when player As’ faced a selfish partner adds novel pharmacogenetic evidence on the ongoing debate over dopamine’s role in appetitive and aversive instrumental learning [4], [44]. While the absence of repayment by player B following a high transfer is associated with monetary loss and is thought to be an aversive social stimulus for player A, positive returns are associated with a monetary gain and are considered to be a rewarding social stimulus [45]. We thus found evidence for a dopaminergic modulation of learning from appetitive, but not from aversive stimuli. This is again in line with dopaminergic drug challenge studies showing a relative selectivity for processing appetitive rather than aversive stimuli in probabilistic learning tasks both in healthy subjects [4], and patients affected with Parkinsońs disease [6].

The probabilistic learning paradigms in non-social contexts employed by research in Parkinsońs disease patients and healthy subjects have some commonality with our task in the sense that investors are required to make stimulus-outcome associations based on probabilistic feedback from the interaction partner. Thus, the fact that the DAT occurs mostly in the striatum, but only in low concentrations in other areas of the brain [14], together with our finding that the DAT1 polymorphism modulates L-DOPA effects on learning about a partners’ prosocial preferences might be an indication that there might also be a common regional neurochemical process at work during reinforcement learning based on social feedback as it is the case for non-social feedback [13]. However, as player As in the current drug study might still have tried to infer player Bś (past) intentions or attitudes to predict repayments when making investments, these inferences might have relied upon a cognitive mentalizing system on top of neural systems such as the reward circuitry [46]. Hence, whether our pharmacogenetic effect is uniquely related to social interactions or rather reflects a relatively broad probabilistic learning mechanism that guides behavior both in social and non-social contexts is a topic for further studies.

## Summary and Conclusions

In sum, while dopaminergic drugs have been shown to affect human reward learning in non-social contexts via modulation of striatal activity, imaging studies of social interactions have shown that activation of the striatum tends to facilitate human cooperative behaviors. Here we show a causal role of the dopaminergic system in learning about otherś prosocial preferences. Exogenous dopamine improves learning performance in subjects who carry the 10/10R genotype, assumed to be associated with lower striatal dopamine levels, and impairs learning performance in people with the 9/10R genotype, assumed to confer higher striatal dopamine levels. These effects might not be related to specifically social situations, but may rather result from a dopaminergic modulation of a general reward learning mechanism. Our findings are not only relevant for fundamental research, but potentially constitute a stepping stone for new ways of understanding psychiatric disorders that link the dopaminergic system with distorted perceptions of otherś prosocial attitudes.

## Supporting Information

Figure S1 Estimated probability of choosing the option with the highest subjective value. (10/10R DAT, placebo: n = 22; L-DOPA: n = 28; 9/10R DAT, placebo: n = 27; L-DOPA: n = 16).
(EPS)

Figure S2 BIC values of the different models employed.
(EPS)

Materials S1. (DOC)
