# Supplementary material for: DAT1 Polymorphism Determines L-DOPA Effects on Learning about Others’ Prosociality
Source: PLoS One. 2013 Jul 4;8(7):e67820. doi: 10.1371/journal.pone.0067820 (PMC3701618; doi:10.1371/journal.pone.0067820)
Supplement: Materials S1. — (DOC) [file pone.0067820.s003.doc]

**SUPPLEMENTARY MATERIAL**

**Reinforcement learning models**

**Q - learning model (QL).** Subjects’ learning about the partners´ prosocial preferences was modeled within the framework of reinforcement learning (RL) [1]. The potential transfers a player A can make define his choice options. The subjective value  of each transfer option  can be thought of as representing the expected return from player B. The probability of choosing a specific choice option is defined by

(1)

where the sensitivity parameter , with 0.01 < < 3, specifies how deterministically player A will choose the option with the highest subjective value. Whereas a large value for implies that the option with the highest subjective value will be chosen with a high probability, a low value for indicates that even options with a low subjective value will be chosen with a substantial probability.

As part of the learning process, the subjective values of the transfers  in trial are updated, using the Rescorla-Wagner delta rule [2]:

(2)

where represents the learning rate, with 0 <   < 1,  * *represents the prediction error, and represents an indicator function that takes the value 1 if transfer was chosen and a value of 0 otherwise. The prediction error is defined as the difference between the subjective value and the actual return

(3)

This way transfers that yield a positive return will lead to an increase in the subjective value associated with that particular transfer. On the other hand, a transfer that leads to an omission of returns will lead to a decrease in the subjective value of that transfer.

**Q - learning model using fictitious prediction errors (QLF).** Since player Bs´ decisions were recorded previously, player As´ transfers cannot influence the likelihood that player Bs will make a back transfer. Player As are informed about this fact and were thus able to calculate the hypothetical returns had they made a different decision in a given trial. For example, a player A who receives a return of 4 MUs following a transfer of 2 MUs can infer that he would have gotten 20 MUs if he had transferred 10 MUs in this round. Thus, player As can determine all potential payoffs in a round using a fictitious prediction error [3,4,5]. Accordingly, player A would update the subjective value of all transfer options and not only of the chosen transfer. To represent this counterfactual learning process, the indicator function of Equation 1 has to take a value of 1 for all transfers.

**Initialization of Q values.** To represent the decision makers’ initial subjective values of the transfers, the initial Q-values were set such that the Q-value for the first chosen transfer was largest and equal to the transferred amount. For example, if the decision maker transferred 5 MU in the first round, the Q-value for a transfer of 5 MU was set to a value of 5. All other transfer options received lower Q-values according to a normal distribution with a mean set at the initially transferred amount *v* and a standard deviation set to the observed mean standard deviation of the first transfer across all participants, which was *SD*_observed_ = 3.22. Accordingly the initial Q-values for all transfer options were determined by

(4)

where represents the probability density function of the normal distribution. We used this procedure to represent the decision maker’s initial preferences for specific transfers. The procedure has the advantage that the predicted learning process only represents how the decision maker changes his initial preferences.

**Parameter estimates and data fitting.** The free parameters of the models were estimated following a maximum likelihood approach. The best parameter values were searched for using a grid-search, with a step size of .01 for and . The grid-search is computationally time demanding, but it guarantees the optimal parameter estimates (by avoiding local maxima problems of other search algorithms). The best parameters minimized the deviance [6]:

(5)

where represents the log-likelihood of the data across all trials given the model and its parameter values.

**Model comparison.** To find the model that is best in describing the observed learning process, we compared the models by their Bayesian information criterion (BIC) [7], which is a model comparison criterion that takes the models’ fit and complexity into account. We determined BIC differences by computing the differences between the BIC value of a baseline model and those of all other models. The baseline model was defined as a model making random choices that chooses each transfer option with a probability of 1/11. A model that does not do better than the naïve baseline model should be considered as an implausible model describing the observed learning process. According to [7], BIC differences above 6 indicate strong evidence and BIC differences above 10 indicate very strong evidence to favor a model compared to the baseline model. The results of the model selection process are shown below (fig. S2).

**Results**

**Quantification of choice sensitivity.** The sensitivity parameter theta of the model has an arbitrary scale. For a more intuitively understandable interpretation of , we express the impact of the sensitivity parameter by reporting the estimated average probability with which a player A chooses a transfer option with the highest subjective value given the estimated sensitivity parameter. This can be derived by averaging the estimated probabilities of choosing the transfer option with the highest subjective value, given the best fitting value of and the respective Q-values (see equation 1). In line with the sensitivity parameter reported in the main text, we found an interaction of L-DOPA and player As’ DAT1 genotype on the estimated probability of choosing the transfer option with the highest subjective value (*F _1,89_ =* 4.399, *P* < 0.039, Figure S1).

***Model selection.*** Average BIC differences indicate very strong evidence of favoring the Q-learning model using fictitious prediction errors (QLF) to the ordinary Q-learning model and the baseline model (Figure S2).

**References**

1. Sutton RS, Barto AG (1998) Reinforcement learning: an introduction. IEEE Trans Neural Netw 9: 1054-1054.

2. Rescorla RA, Wagner AW (1972) A theory of Pavlovian conditioning: Variations in the effectiveness of reinforcement and nonreinforcement. In: Black AH, Prokasy WF, editors. Classical Conditioning II: Current Research and Theory: Appleton-Century-Crofts. pp. 64-99.

3. Büchel C, Brassen S, Yacubian J, Kalisch R, Sommer T (2011) Ventral striatal signal changes represent missed opportunities and predict future choice. NeuroImage 57: 1124-1130.

4. Camerer CF (2003) Behavioral game theory experiments in strategic interaction. Princeton, N.J.: Princeton University Press.

5. Gläscher J, Hampton AN, O'Doherty JP (2009) Determining a role for ventromedial prefrontal cortex in encoding action-based value signals during reward-related decision making. Cereb Cortex 19: 483-495.

6. Lewandowsky S, Farrell S (2011) Computational modeling in cognition: principles and practice. Thousand Oaks, CA: SAGE.

7. Raftery A (1995) Bayesian model selection in social research. Soc Method 25: 111-163.
